# Evaluating measurement properties of subjective cognitive decline self-reported outcome measures: a systematic review

**DOI:** 10.1186/s13643-022-02018-y

**Published:** 2022-07-18

**Authors:** Aliaa Ibnidris, Janelle N. Robinson, Marissa Stubbs, Giovanni Piumatti, Ishtar Govia, Emiliano Albanese

**Affiliations:** 1 Faculty of Biomedical Sciences, Università della Svizzera italiana, Lugano, Switzerland; 2 Department of Psychiatry and Mental Health, University of Cape Town, Cape Town, South Africa; 3 Epidemiology Research Unit, Caribbean Institute for Health Research, The University of the West Indies, Mona Campus, Kingston, Jamaica; 4 Fondazione Agnelli, Turin, Italy

**Keywords:** Subjective, Cognitive dysfunction, Preclinical AD, Measurement properties, Assessment, PROM

## Abstract

**Background:**

Subjective cognitive decline (SCD) is present in the early stage of preclinical Alzheimer’s disease (AD) and is associated with an increased risk of further cognitive decline and AD dementia later in life. Early detection of at-risk groups with subjective complaints is critical for targeted dementia prevention at the earliest. Accurate assessment of SCD is crucial. However, current measures lack important psychometric evaluations and or reporting.

**Objectives:**

To systematically evaluate measurement properties of self-reported outcome measures (PROMs) used to assess SCD in the older adult population with or at risk of AD.

**Methods and analysis:**

We used the Preferred Reporting Items for Systematic Reviews and Meta-Analysis Protocols 2015 Checklist for reporting. We conducted a literature search, screened, and included validation studies of SCD based on self-reported questionnaires from both population-based and clinical studies, conducted in older adults (≥ 55). We critically appraised the included primary studies using the Consensus-based Standards for the selection of health Measurement Instruments (COSMIN) guidelines.

**Results:**

Sixteen studies met the inclusion criteria. The included studies reported psychometric properties of 17 SCD self-reported questionnaires. We extracted data on the structural validity, internal consistency, test-retest reliability, and cross-cultural validity and found a widespread proneness to bias across studies, and a marked heterogeneity is assessed and reported measurement properties that prevented the consolidation of results.

**Conclusion:**

Our findings suggest that available SCD questionnaires lack content validity evaluation. Currently available measurements of SCD lack development and validation standards. Further work is needed to develop and validate SCD self-reported measurement with good quality measurement properties.

**Supplementary Information:**

The online version contains supplementary material available at 10.1186/s13643-022-02018-y.

## Introduction

Targeted dementia prevention requires the early detection and diagnosis of at-risk individuals of Alzheimer’s disease (AD) dementia. Several biomarkers (e.g., amyloid plaques and neurofibrillary tangles) are present in the brain many years before dementia develops [1]. Furthermore, neuronal damage and loss may already occur in earlier stages such as mild cognitive impairment (MCI), leading to irreversible cognitive dysfunction [2]. The US National Institute on Aging – Alzheimer’s Association (NIA-AA) proposed a preclinical stage of AD characterized by normal cognitive performance in standardized neuropsychological tests and the presence of AD biomarkers [3–6]. This stage may be accompanied by a subtle cognitive decline that is only perceived subjectively but not captured by standardized tests [7]. Therefore, detection at this stage is of particular interest for AD prevention, including disease-modifying trials [7, 8].

Subjective cognitive decline (SCD) is defined as a self-perceived, progressive decline in cognitive abilities such as memory, executive functions, or language [7]. Previous evidence suggests that older adults with SCD have an increased risk of further cognitive decline and conversion to MCI or AD dementia in late life [9–12]. SCD as a separate construct has been gaining more attention and is being suggested to be one of the earliest symptoms in the preclinical stage of AD [7, 13]. The Subjective Cognitive Decline Initiative (SCD-I) working group defined key concepts of SCD and propose it as a symptomatic stage of preclinical AD [7]. However, assessment of SCD varies greatly between studies, and standardization and operationalization are lacking [7]. Recommendations from the SCD-I working group include a thorough evaluation of psychometric properties of available self-reported measures used in the current literature [14]. SCD is commonly assessed using a single-item approach (e.g., “Do you feel that your memory is getting worse?”) with a dichotomous response option (yes/no) [15–17]. This approach does not cover two important aspects of SCD. First, self-perceived decline can affect other cognitive domains than memory. By asking about memory alone, potential perceived failures in other domains (e.g., executive function or language) may be overlooked. Second, it is important to ask about the time of onset and graduality of the subjective decline. A gradual perceived change in cognitive function over recent years is more likely to be an early manifestation of AD than the persistent feeling of change that has been present for many years [7, 18, 19]. Another aspect to consider is the inclusion of an informant’s report. The proposed diagnostic criteria for SCD by the SCD-I recommends including an informant’s report of cognitive changes of the older adult. While several SCD PROMs are currently used in research and clinical studies, the methodological quality of these measures varies greatly [14]. The importance of reliability and validity, two of the core psychometric properties in scale development, lies in the ability to capture subjective decline in cognitive functions accurately and consistently. To the best of our knowledge, our study is the first systematic review to evaluate the psychometric properties of available self-reported questionnaire that are used to assess SCD in older adults.

Building on the recommendation of the SCD-I, the main aim of this work was to conduct a systematic review to evaluate the psychometric properties of self-reported output measures used to assess SCD in older adults with or at risk of AD. The research question follows the Consensus-based Standards for the selection of health Measurement Instruments (COSMIN) format [20–22]:

- The construct or the name(s) of the outcome measurement instrument(s) of interest: SCD in AD
- The target population: older adults 55 years old and above
- The type of measurement instrument of interest: self-reported questionnaires used to assess SCD in older adults in the context of AD
- The measurement properties on which the review focuses: structural validity, internal consistency, test-retest reliability, and cross-cultural validity

## Methods

The protocol of this systematic review was registered on PROSPERO (CRD 42020166905). The protocol and the systematic review were reported following the Preferred Reporting Items for Systematic reviews and Meta-Analyses (PRISMA) guidelines [23, 24].

### Study selection

We attempted to identify original studies that reported PROM development to assess SCD in older adults in the context of Alzheimer’s disease. We included studies that performed and reported validation of psychometric properties, specifically on the validity and reliability of SCD PROMs. We included community-based studies as well as studies conducted in memory clinics or research settings. We excluded studies that used SCD PROMs to recruit participants for specific studies or studies that did not aim to validate PROMs. We also excluded studies that developed and validated SCD PROMs for the purpose of screening or diagnosing SCD in other diseases (e.g., depression).

### Inclusion criteria

#### Participants

We included studies with older adults (55 years and older) in studies of Alzheimer’s disease (including studies about Mild Cognitive Impairment and AD dementia studies). We attempted to include studies that tested SCD PROMs in cognitively healthy adults as well as adults diagnosed with SCD, MCI, or AD dementia. We excluded studies with older adults with SCD due to any other specific, previously identified conditions such as stroke, neuropsychiatric conditions (i.e., mood disorders, psychotic disorders), trauma, delirium, or disability.

#### Time frame

We included relevant studies published between 1982 and 2020 that were published in English. We chose the year 1982 because the concept of SCD was first described in 1982 [25, 26].

### Data sources

We searched for published studies using the main and most relevant biomedical databases to our study focus:

- MEDLINE/PubMed
- Embase
- PsycINFO

For gray literature, we used the OpenGrey database. To look for thesis and dissertations, we used Open Access Theses and Dissertations and WorldCat databases. For conference proceedings and abstracts, we used Web of Science and Scopus. Studies included in the review were not limited to a certain geographical location.

### Search strategy

Our search strategy included iterations of the concepts Subjective Cognitive Decline AND Preclinical Alzheimer’s disease AND self-reported questionnaire AND measurement properties. The full search strategy is available in Additional file 1.

### Study records

- *Data management:* We stored all records, articles, and related material using OneDrive. We used Zotero for bibliographic management for all retrieved studies and to remove duplicates.
- *Selection process:* Three independent reviewers (AI, JR, and MS) screened the study titles and abstracts against the eligibility criteria. The first reviewer (AI) then imported the selected studies to Rayyan—a web-based software for the title and abstract screening in systematic reviews [27]—for the title and abstract screening phase. Any conflict between the three reviewers was resolved through discussion. Full texts of the included records were independently reviewed by the three reviewers to determine the eligibility for data extraction and analysis. This was followed by another session to resolve discrepancies between the three reviewers. In case of unresolved conflict, a senior researcher (EA) was consulted to make the final decision.
- *Data collection process:* The first reviewer (AI) abstracted the data from full records independently using the COSMIN data abstraction tables. When full articles were not available, we contacted the corresponding authors and requested the full text. The three reviewers (AI, JR, and MS) completed the risk of bias checklist and rating of the quality of measurements using the COSMIN material.

### Main outcomes

We used the COSMIN methodology in a modular manner to evaluate any reported psychometric property in the included study. However, for the purpose of this systematic review, we will focus on the internal structure of PROMs and qualitatively analyze the structural validity, reliability (internal consistency and test-retest reliability), and cross-cultural validity. The main outcomes are the following measurement properties:

1. Content validity
  - Self-reported outcome measurement development
  - Content validity evaluation
2. Internal structure
  - Structural validity
  - Internal consistency and test-retest reliability
  - Cross-cultural validity

Because there is no gold standard measurement to assess SCD, we ignored the results on criterion validity in the included studies and did not include them in the analysis. Furthermore, a number of the included studies also reported construct validity (convergent and concurrent validity) of the SCD PROM, however with clinically administered assessment measures of cognitive decline (e.g., MMSE). We ignored these results as well because construct validity should be tested with other validated measures that assess SCD.

### Data extraction

Data extraction was conducted independently by three reviewers. We used the developed extraction sheet by COSMIN and piloted it at the start of data extraction. We extracted the following information variables:

- The characteristics of the self-reported outcome measurement include the name of the measure, a reference to the article in which the development of the measure is described, constructs being measured, language and study population for which the measure was developed, intended context of use, the available language version of the measure, number of items in each scale, and response options.
- Characteristics of the included samples include geographical location, language, target population, sample size, percentage of female participants, and mean age of the study sample.
- Methodological quality ratings per measurement property per PROM.

### Measures of effect

Each measurement property is evaluated by rating the relevant sub-items listed below. For example, in internal consistency, if the validation study of the PROM reports that Cronbach’s alpha was calculated for continuous scores, then this sub-item is rated “very good.” If not, it is rated “doubtful” or “inadequate” depending on which other statistical tests were performed. The measures of effects per psychometric property are presented in Table 1.

**Table 1:** Outcome measures per measurement properties

Measurement property	Measure(s) of effect
**Self-reported outcome measurement development**	Concept elicitation: sample size (evaluated based on COSMIN guidelines) [20, 22]
	Cognitive interview study: number of patients per item
	Comprehensiveness: number of patients per item
**Content validity**	Relevance (patients): number of patients per item
	Comprehensiveness (patient): number of patients per item
	Comprehensibility (patient): number of patients per item
	Relevance (professionals): number of professionals per item
**Structural validity**	Exploratory factor analysis (EFA)
	Confirmatory factor analysis (CFA)
**Internal consistency**	Continuous scores: Cronbach’s alpha or omega
	Dichotomous scores: Cronbach’s alpha or KR-20
	Item response theory (IRT)-based scores: SE (*θ*) or reliability coefficient
**Reliability**	Continuous scores: intraclass correlation coefficient (ICC)
	Dichotomous/nominal/ordinal scores: kappa, weighted kappa (ordinal scores)
**Cross-cultural validity**	Whether the PROM shows similar structural validity and reliability when validated in another cultural context or translated to another language

### Risk of bias

We evaluated the risk of bias using the COSMIN Risk of Bias Checklist [20–22]. The checklist assesses the quality of the relevant main outcomes described above. Each psychometric property is evaluated by scoring a set of items about the conduction and reporting of the specific property. Each item is scored either “V” (very good), “A” (adequate), “D” (doubtful), or “I” (inadequate) according to the instruction of the COSMIN rating guideline. The total score for each psychometric property is given based on the “worst score count” method. The COSMIN guidelines instruct the raters to rate the overall quality of a property by taking the lowest rating given to any of the sub-items. The risk of bias per psychometric property per PROM was evaluated by the three reviewers (AI, JR, and MS). Any discrepancy between the three reviewers was discussed to reach a consensus.

### Strategy for data synthesis

We did not conduct a meta-analysis, and we only described the quality of psychometric properties testing in the selected studies. We used the COSMIN criteria to evaluate each psychometric property that was tested and reported for each SCD PROM. We evaluated validity (structural validity) and reliability (internal consistency and test-retest reliability) properties. We further evaluated cross-cultural validity and convergent validity when possible. We evaluated each reported psychometric property per PROM before judging its risk of bias. Based on the statistical analysis and reported results of each psychometric property, we also provided a qualitative assessment on whether the results are “sufficient,” “indeterminate,” or “insufficient.”

### Analysis of subgroups or subsets

We did not conduct an analysis of subgroups or subsets in this review.

### Confidence in cumulative evidence

The COSMIN guidelines recommend using the modified version of the GRADE approach (Grading of Recommendations Assessment, Development and Evaluation) to grade the strength and quality of the collected evidence. Because the identified studies did not have content validity studies, we were not able to grade the selected SCD PROM studies using the modified GRADE approach.

## Results

### Results of the search

We completed the search on 10 June 2021. We identified 364 records in the initial database search. We further identified 13 records through hand-searching the references of some of the relevant studies. After duplicate removal, there were 290 records remaining. The three reviewers (AI, JR, and MS) screened the title and abstract of the 290 records using Rayyan software. After the completion of the screening phase, the inter-rater reliability and agreement between the three reviewers were measured using Fleiss kappa [28] and agreement percentage in RStudio [29]. The results indicate substantial agreement between the reviewers (Fleiss kappa = 0.662, *p*-value < 0.0001). Moreover, the percentage agreement with zero tolerance was 78.4%. After the resolution of the conflict between the three reviewers, we excluded 208 records that did not meet the inclusion criteria and 10 records that did not have the full text available (conference proceedings). One further duplicate study was identified at the stage of full-text review. We screened the full texts of the remaining 71 records.

### Included studies

**Fig. 1:**
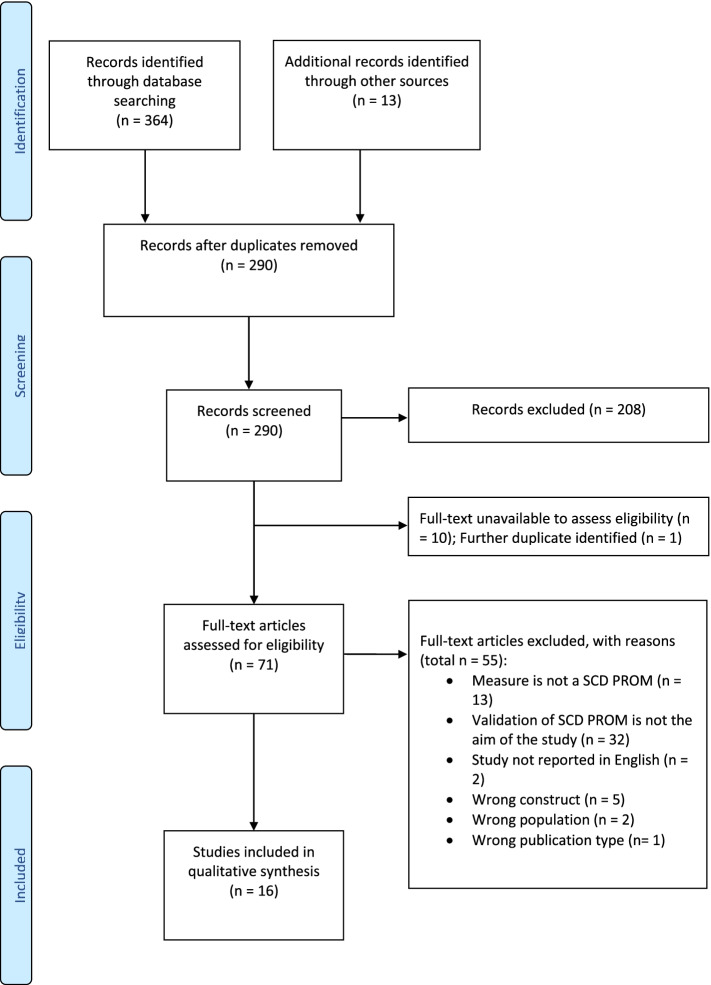
Flow chart of the screening and selection process of the identified records

The final number of included studies was sixteen studies. The search and selection of studies are demonstrated in a PRISMA flow chart (Fig. 1). All studies developed and validated SCD PROMS in high-income settings, mainly in the USA (37.5%) and Europe (56.25%). Only one study was conducted in Asia (South Korea). Table 2 summarizes the characteristics of the included studies that reported measurement properties of SCD self-reported questionnaires in older adults. Additional file 1: Table S3 provides an overview of excluded studies after full-text review, with an explanation on the reasons to exclude as well as citations (available in the supplementary material).

**Table 2:** Details and characteristics of the studies included in the systematic review

First author (year)	Location	Sample size	Study population	Mean age (SD)	Female (%)	Study design	Setting	Language of development	Language of validation
**Allison et al. (2019) **[30]	USA	91	Cognitively healthy adults	69 (6)	54 (59.34)	Cross-sectional	Laboratory	English	English
**Avila-Villanueva et al. (2016) **[31]	Spain	844	Cognitively healthy adults (*n* = 766); MCI (*n* = 78)	Cognitively healthy = 74.07 (3.80); MCI = 76.08 (4.06)	Cognitively healthy = 63% female; MCI = 50%female	Longitudinal	Research setting (unspecified)	English	Spanish
**Chipi et al. (2018) **[32]	Italy	257	Cognitively healthy adults	70.9 (5.1)	158 (61.2%)	Cross-sectional	Memory clinic	English	Italian
**Crook et al. (1992) **[33]	USA	232	Cognitively healthy adults	59.2 (6.9)	117 (50.43%)	Clinical trial	Unspecified	English	English
**Crowe et al. (2006) [**34]	USA	55	MCI	76 (NA)	39 (71%)	Longitudinal	Unspecified	English	English
**Crowe et al. (2006) [**34]	USA	55	MCI	76 (NA)	39 (71%)	Longitudinal	Unspecified	English	English
**Gifford et al. (2015) **[35]	USA	191	Cognitively healthy adults (*n* = 115); MCI (*n* = 43); Other (n =33)	Cognitively healthy = 75.9 (7.5); MCI = 77.0 (6.5); others = 78.5 (8.5)	Cognitively healthy = 72.24 (63%); MCI = 21.9 (51%); others = 19.14 (58%)	Longitudinal	Unspecified	English	English
**Gilewski et al. (1990) **[36]	USA	778	Cognitively healthy adults	56.9 (20.8)	435 (55.91%)	Cross-sectional/longitudinal	Unspecified	English	NA
**La Joie et al. (2016) **[37]	France	185	Cognitively healthy (*n* = 74); MCI (*n* = 78); SCD (*n* = 33)	Cognitively healthy = 69 (7.2); MCI = 73 (7.2); SCD = 68 (7.3)	Cognitively healthy = 40 (54%); MCI = 38 (49%); SCD = 14 (42%)	Cross-sectional	Memory clinic	English	French
**Lubitz et al. (2018) **[38]	Germany	734 above 65 = 83.67 (11.4%)	Cognitively healthy adults	43.15 (17.17);	401 (62.26%) of subsample of 644 participants	Longitudinal	Community-based	German	German
**Papaliagkas et al. (2017) **[39]	Greece	295 older adults (*n* = 53) Older-old adults (*n* = 28)	Cognitively healthy adults	Older adults = 69.9 (3.6) Older-old adults = 83.5 (3.3)	Older adults = 31 (56.6%) Older-old adults = 18 (64.3%)	Cross-sectional	Unspecified “quiet and comfortable environment”	English	Greek
**Papaliagkas et al. (2017) **[39]	Greece	295 older adults (*n* = 53) Older-old adults (*n* = 28)	Cognitively healthy adults	Older adults = 69.9 (3.6) Older-old adults = 83.5 (3.3)	Older adults = 31 (56.6%) Older-old adults = 18 (64.3%)	Cross-sectional	Unspecified “quiet and comfortable environment”	English	Greek
**Rami et al. (2014) **[40]	Spain	397	Cognitively healthy (*n* = 124); MCI (*n* = 83), AD (*n* = 46); SCD (*n* = 144)	Cognitively healthy = 60.2 (9.2); P-SCD = 56.8 (10.4); C-SCD = 67.0 (8.9); MCI = 70.5 (9.2); AD = 74.5 (8.8)	Cognitively healthy = 66 (59%); P-SCD = 33 (58%); C-SCD = 58 (71%); MCI = 39 (49%); AD = 29 (64%)	Cross-sectional	Unspecified	Spanish	Spanish
**Rattanabannakit et al. (2016) **[41]	USA	267	Cognitively healthy (*n* = 149); MCI (96); AD (*n* = 22)	67.8 (11.2)	138 (51.7)	Cross-sectional	Memory clinic	English	English
**Valech et al. (2018) **[42]	Spain	68	Cognitively healthy (*n* = 37); SCD (*n* = 31)	Cognitively healthy = 64.49 (6.89); P-SCD = 59.33 (5.82); C-SCD = 65.72 (7.02)	Cognitively healthy = 23 (62.2%); P-SCD = 6 (100%); C-SCD = 17 (68.0%)	Cross-sectional	Memory clinic/home	Spanish	Spanish
**Vestergren et al. (2011) **[43]	Sweden	370	Cognitively healthy adults	65 (15)	194 (52.7)	Longitudinal	Home	Swedish	Swedish
**Vestergren et al. (2012) **[44]	Sweden	1115	Cognitively healthy adults	63.0 (14.5)	598 (53.7)	Longitudinal	Unspecified	Swedish	Swedish
**Youn et al. (2009) **[45]	South Korea	1651	Cognitively healthy (*n* = 1464); AD (*n* = 187)	74.3 (8.2)	945 (57.3)	Longitudinal	Unspecified	Korean	Korean

*Abbreviations*: *AD* Alzheimer’s disease, *MCI* mild cognitive impairment, *SCD* subjective cognitive decline, *P-SCD* SCD sample from the population, *C-SCD* SCD sample from clinical settings

### Participants

Fifteen studies validated the questionnaires in a sample including both cognitively healthy older adults as well as adults diagnosed with SCD, MCI, or AD. Seven included studies developed and validated SCD questionnaires only in cognitively healthy older adults [30, 32, 36, 38, 39, 43, 44]. One study included participants with MCI only [34]. Older adults with MCI were further included in 6 of the included studies [31, 33, 35, 37, 40, 41]. Only two studies included participants who received a prior diagnosis of SCD [37]. Participants with clinically diagnosed AD dementia were included in two studies [40, 45].

### Assessed cognitive domains

Sixteen of the seventeen SCD PROMs included items to assess subjective decline in memory function. Nine PROMs looked only at the memory and included different aspects of memory aspects such as facial recognition, spatial topographic memory, word and fact recall/semantic memory, general forgetfulness, everyday task-oriented memory, numeric recall, and remote personal memory [33]. Other PROMs focusing on memory aimed to assess retrospective and prospective memory [37, 39] and episodic memory [32] or to capture self-perceived change in memory functioning overall [34]. One PROM was developed to assess subjective decline in spatial navigation skills only [30]. Six PROMs assess subjective complaints in executive functions and praxis [31, 32, 37, 38, 40–42]. Self-perceived decline in language abilities was assessed only in three PROMs [37, 40–42]. Lastly, social cognition was assessed in one PROM [32].

### Reported psychometric properties

As shown in Table 2, of the included studies, 81.25% performed and reported aspects of the PROM development procedure (*n* = 13) [30–36, 39, 40, 43, 45] 75% (*n* = 12) of which reported conducting a cognitive interview or piloting the generated pool of questionnaire items to assess comprehensibility and comprehensiveness of the selected items [30–38, 40, 43, 45]. However, all studies but one [38] did not report details regarding the piloting phase. None of the included studies tested for content validity of the developed PROM. On the other hand, the majority of studies tested and reported structural validity (75%, *n* = 12) for thirteen PROMs [30, 31, 35–40, 42–45]. .Four of which (25%) used exploratory factor analysis (EFA) only [30, 31, 37, 42], while two studies used EFA followed by confirmatory factor analysis (CFA) [35, 36]. Four studies only reported and/or conducted CFA only [38, 39, 44, 45]. Internal consistency using Cronbach’s alpha was reported in 68.75% of studies (*n* = 11) for 13 PROMs [30, 32–34, 36, 38–41, 43–45]. One study used item response theory (IRT) to test for internal consistency and reported the standard error (SE *θ*) [35]. One study reported that internal consistency was tested; however, no statistic was provided [31]. Test-retest reliability was reported in four studies [30, 33, 36, 45]. Only four studies (25%) indicate cross-cultural validation of SCD PROMs in other languages [31, 32, 37, 39]. Two studies evaluated and reported convergent validity with other SCD PROMs [33, 43].

### Risk of bias

All twelve studies that reported PROM development steps received “I” rating (i.e., high risk of bias) due to the lack of conducting or reporting a cognitive interview or asking about the comprehensibility or comprehensiveness of the PROM items. Seven of the thirteen PROMs performed CFA and received a “V” rating in structural validity testing, therefore had a low risk of bias [36, 38–40, 44, 45]. One study was rated “A” for structural validity and was judged to have a moderate risk of bias [31]. The remaining five PROMs were judged to have a high risk of bias due to having an inadequate sample size and receiving “I” rating on the reported structural validity [30, 35, 38, 42, 43].

Regarding internal consistency, of the 15 PROMs that were tested for internal consistency studies, 12 were judged to have a low risk of bias (“V” rating) [34–36, 38–41, 43–46]. One PROM, the spatial navigation questionnaire was judged to have a moderate risk of bias (“A” rating) [30]. The EMQ and the CFI both had a D rating, i.e., show a high risk of bias [31, 32]. The remaining PROMs were judged to have a high risk of bias as well (“I” rating) [37, 42].

Moreover, two PROMs, the spatial navigation questionnaire and the MFQ [30, 36], had a V rating for the test-rest reliability and were therefore of low risk of bias. On the other hand, the MAC-Q and the SMCQ were of high risk of bias (“D” rating) [33, 45]. All four PROMs that evaluated cross-cultural validity of the translated version of the questionnaire were of low risk of bias (“V” rating) [31, 37, 39]. The risk of bias evaluation is available in Additional file 1: Table S1.

### Included SCD PROMS and the quality of the reported psychometric properties

The included studies evaluated 17 PROMs in total that are used to assess SCD in adults. Table 3 provides details of the validated SCD PROMS, which cognitive domains are covered, and the response options and their scoring system. The results of the reported psychometric property per PROM as well as a summary of the quality of each measurement property of the abovementioned SCD PROMs, as rated against the COSMIN criteria of good measurement property, are available in the appendix (in Additional file 1: Tables S2–S3). The focus of each PROM is elaborated below.

**Table 3:** Characteristics of SCD PROMS in the included studies

First author (year)	PROM	Domain	Target population	Reported purpose	Informant	Items	Response	Scoring	Mode of administration	Time to administer (min)
**Allison et al. (2019) **[30]	Spatial Navigation Questionnaire	Spatial navigation	Older adults	Screening	Yes	20	7-point Likert scale	NA	Self-administered: Electronic	5
**Avila-Villanueva et al. (2016)** [31]	Everyday Memory Questionnaire	Forgetfulness of immediate information, executive functions, prospective memory, forgetfulness of common objects, and spatial orientation	Older adults	Discriminative	No	28	3-point Likert scale	0–56	Unspecified	Not reported
**Chipi et al. (2018)** [32]	Cognitive Function Instrument	Memory, prospective memory, episodic memory, executive functions, spatial orientation, and social cognition	Older adults	Diagnosis, follow-up	Yes	14	Yes; maybe; no	0–14	Unspecified	Not reported
**Crook et al. (1992)** [33]	Memory Complaint Questionnaire (MAC-S)	Memory (facial recognition, spatial topographic memory, word and fact recall/semantic memory, general forgetfulness, everyday task-oriented memory, numeric recall, remote personal memory, and attention/concentration)	Older adults	Screening	No	49	5-point Likert scale	7-35	Unspecified	Not reported
**Crowe et al. (2006) [**34]	Attitude Toward Intellectual Aging Scale of the Personality in Intellectual Aging Contexts (PIC) Inventory (six items)	Change in memory	Older adults	Screening	No	6	6-point Likert scale	Not reported	Unspecified	Not reported
**Crowe et al. (2006) [**34]	Short version of Memory Functioning Questionnaire (MFQ)	Memory	Older adults	Screening	No	14	7-point Likert scale	Not reported	Unspecified	Not reported
**Gifford et al. (2015) **[35]	SCD questions	Global memory functioning, temporal comparisons	Older adults	Screening	No	9	Yes/no (6 items), 3-point Likert scale (3 items)	Unclear	Self-administered: mail-in	Not reported
**Gilewski et al. (1990) **[36]	Memory Functioning Questionnaire (MFQ)	Memory	Older adults	Screening	No	64	7-point Likert scale	Unclear	Unspecified	Not reported
**La Joie et al. (2016) **[37]	Cognitive Difficulties Scale	Retrospective and prospective memory, attention, language, orientation, and praxis	Older adults	Screening	No	39	5-point Likert scale	Unclear	Unspecified	Not reported
**Lubitz et al. (2018) **[38]	Complainer Profile Identification	Memory, attention, and executive function	Older adults	Monitoring	No	17	5-points Likert scale	Unclear	Self-administered: pen-and-paper; electronic/online	Not reported
**Papaliagkas et al. (2017) **[39]	Cognitive Failures Questionnaire (CFQ)	General cognitive functions	Older adults	Screening	No	25	5-point Likert scale	Unclear	Unspecified	Not reported
**Papaliagkas et al. (2017) **[39]	Prospective and Retrospective Memory Questionnaire (PRMQ)	Prospective and retrospective memory	Older adults	Screening	No	16	5-point Likert scale	Unclear	Unspecified	Not reported
**Rami et al. (2014) **[40]	The Subjective Cognitive Decline Questionnaire (SCD-Q)	Memory, language, and executive functions	Older adults	Diagnosis	Yes	24	Yes/no	0–24	Self-administered	Not reported
**Rattanabannakit et al. (2016) **[41]	Cognitive Change Index (CCI)	Memory, executive function, and language	Older adults	Screening	Yes	20	5-point Likert scale	Unclear	Self-administered: pen-and-paper/mail-in	Not reported
**Valech et al. (2018) **[42]	The Subjective Cognitive Decline Questionnaire (SCD-Q)	Memory, language, and executive functions	Older adults	Diagnosis	Yes	24	Yes/no	0–24	Self-administered: At home	Not reported
**Vestergren et al. (2011) **[43]	Cognitive Dysfunction Questionnaire (CDQ)	Memory (working memory, semantic memory, episodic memory, procedural memory, prospective memory) and global cognitive (spatial navigation, temporal orientation)	Older adults	Screening	No	20	5-point scale: 1 to 5 (very seldom = 1, very often = 5)	Unclear	Self-administered: at home	Not reported
**Vestergren et al. (2012) **[44]	Cognitive Dysfunction Questionnaire (CDQ): refined version	Memory (working memory, semantic memory, episodic memory, procedural memory, prospective memory), global cognitive (spatial navigation, temporal orientation) + procedural actions, semantic word knowledge, face recognition, temporal orientation, spatial navigation, and episodic memory	Older adults	Screening	No	40	5-point scale: 1 to 5 (very seldom = 1, very often = 5)	Unclear	Unspecified	Not reported
**Youn et al. (2009) **[45]	Subjective Memory Complaints Questionnaire (SMCQ)	Global memory	Older adults	Screening	No	14	Yes/no	14	Unspecified	Not reported

The spatial navigation questionnaire is a self-report questionnaire that assesses self-perceived decline in spatial navigation skills over the past several years [30]. It includes self- and informant reports, and each part consists of 20 items of statements regarding the change in navigation abilities. The response options range from 1 (strongly disagree) to 7 (strongly agree). The questionnaire was validated in a laboratory setting with a sample of 91 cognitively healthy older adults (mean age = 69, SD = 6). The questionnaire was administered to participants and their informants in two visits, 3 months apart. The authors report using exploratory factor analysis (EFA) to identify 20 out of 30 initial items that measure self-perceived change in navigation skills. However, due to deploying EFA only, the quality of the structural validity was deemed “indeterminate.” Internal consistency for the participant’s form was sufficient (Cronbach’s *α* = 0.965, CI = 0.953–0.974), and for the informant’s form (Cronbach’s *α* = 0.957, CI = 0.942–0.970) as well as test-rest reliability for the participants (ICC = 0.838, CI = 0.743–0.900) and the informant part (ICC = 0.723, CI = 0.552–0.835) [30].

Everyday Memory Questionnaire (EMQ) is a 25-item questionnaire that comprises a five-factor structure [31]: (1) forgetfulness of immediate information (FII) was associated with fails in immediate retrieval as well as naming impairment; (2) executive functions; (3) prospective memory (PM); (4) forgetfulness of common objects (FCO); and (5) spatial orientation (SO). The PROM was validated in a sample of 844 participants, 766 of which were cognitively healthy controls (90.8%) and 78 had MCIs (9.2%). The mean age for cognitively healthy participants was 74.07 (SD = 3.80) and 76.08 (SD = 4.06) for participants with MCI. Items were scored on a 3-point scale, with 0 representing “never, rarely,” 1 “occasionally, sometimes,” and 2 “frequently, almost always.” The total score ranged from 0 to 56. The authors report testing for structural validity (EFA) and internal consistency. However, no statistical test for internal consistency was reported. Cross-cultural validity is implicitly indicated because the EMQ was translated from English to Spanish and was validated in a Spanish-speaking population.

The cognitive functions instrument (CFI) contains 14 questions and has two parts, a participant and an informant’s form [32]. It assesses the presence of subjective cognitive concerns in older adults. The response options are yes = 1, maybe = 0.5, and no = 0, and the total score of the questionnaire ranges from 0 to 14. The CFI was validated in the Italian language in a sample of (mean age = 70.9, SD = 5.1). The authors report a more detailed and thorough translation and cultural adaptation process which indicated sufficient cross-cultural validity. The CCFI was only evaluated for the internal consistency for the participant’s (Cronbach’s *α* = 0.77, 95% CI = 0.72–0.83) as well as the informant’s part (group 1: Cronbach’s *α* = 0.77, 95% CI 0.70–0.85; group 2: Cronbach’s *α* = 0.72, 95% CI = 0.66 to 0.78) [32].

The Memory Complaint Questionnaire (MAC-Q) is a 6-item self-report questionnaire that assesses age-associated memory decline in older adults in a clinical trial for experimental treatment of age-associated cognitive decline [33]. The response options are either yes or no, and the total score ranges from 7 to 35. The authors reported that MAC-Q was administered again after 12 weeks to assess test-retest reliability; however, the reported statistic was not specified. The reported internal consistency of the MAC-Q was not sufficient (Cronbach’s *α* = 0.57). The study also evaluated the convergent validity of another validated SCD questionnaire, the MAC-S. Note that the authors referred to this as “concurrent validity.” The Memory Assessment Clinics-Self-rating (MAC-S) is a 49-item memory questionnaire [46]. Twenty-one items assess the person’s ability to remember specific types of information, and 24 items evaluate how often specific memory problems are experienced. The last 4 items ask about the person’s overall assessment of his or her memory. The MAC-S assesses different memory skills (e.g., facial recognition, spatial topographic memory, word and fact recall). The response options are rated on a 5-point Likert scale and the total score of the 49 items. The reported concurrent validity was sufficient (*r* = 0.41, *p*-value < 0.001).

One study reported measurement properties of two different PROMs. The first was a set of six items extracted from the Attitude Toward Intellectual Aging Scale of the Personality in Intellectual Aging Contexts (PIC) Inventory [47]. The response options ranged from 1 (strongly agree) to 6 (strongly disagree) and were summed for the overall score. The second PROM is the General Frequency of Forgetting Scale of the Memory Functioning Questionnaire (MFQ) [36]. The subscale of the MFQ has 14 items that assesses the change in memory. Response options ranged from 1 (always) to 7 (never) and were summed to obtain the overall score. For both scales, higher scores reflected a greater perception of cognitive decline. The authors report sufficient internal consistency for both scales (Cronbach *α* = 0.81 and 0.92, respectively).

Gifford et al. [35] developed a scale of 9 items to assess SCD using EFA followed by confirmatory factor analysis (CFA) to evaluate the unidimensionality of the scale, as well as item-response theory (IRT) to test for internal consistency. Participants were mailed a questionnaire containing 57 items that ask about everyday memory failures. In the final version, the response options were dichotomous (yes/no) for the first 6 questions. For the remaining questions, the responses were rated on a 3-point Likert scale, ranging from “always,” “sometimes,” or “never a problem.” The final version of the 9-item questionnaire shows sufficient structural validity (CFA was performed) and internal consistency (the mean SE (*θ*) score was 20.12–0.90 for cognitively healthy participants and 0.34–0.83 for participants with MCI).

The Memory Functioning Questionnaire (MFQ) was developed and validated in 778 cognitively healthy older adults [36]. It contains 64 items that assess subjective memory worsening. The items are rated on a 7-point Likert scale that ranges from. Structural validity was tested by EFA followed by CFA. The final version of 64 items consists of four factors: (1) general frequency of forgetting, (2) seriousness of forgetting, (3) retrospective functioning, and (4) mnemonics usage. Reported Cronbach *α* for internal consistency of each factor was 0.94, 0.94, 0.89, and 0.83, respectively. The authors refer to test-retest reliability being measured; however, no ICC or weighted kappa was calculated, and therefore, test-retest reliability was rated “insufficient.”

The Cognitive Difficulties Scale (CDS) is a 39-item questionnaire that assesses how often someone is currently experiencing cognitive difficulties in everyday life [37, 48]. The items are rated on a 5-point scale, ranging from “never” = 0 to “very often” = 4. The authors of the included study validated the SCD in the French language in a sample of 185 older adults (mean age: cognitively healthy = 69, SD = 7.2; MCI = 73, SD = 7.2; SCD = 68, SD = 7.3). The reported structural validity assessment was rated “inadequate” due to the sample size.

The complainer profile identification (CPI) is a 17-item scale that assesses subjective change in memory, attention, and executive function [38]. It was validated in a sample of 734 German adults, 83.67 (11.4%) of which were above 65 years old (mean age = 43.15, SD = 17.17). The items are rated on a 5-points Likert scale, ranging from “never” = 0 to “very often” = 4. Both structural validity (CFA) and internal consistency (Cronbach’s *α* = 0.87) were sufficient as rated by the criteria COSMIN of good measurement property.

Papaliagkas et al. [39] reported structural validity, reliability, and cross-cultural validity of the Greek versions of the Cognitive Failures Questionnaire (CFQ) [49] and the Prospective and Retrospective Memory Questionnaire (PRMQ) [50]. Both scales were translated and validated in a sample of 295 Greek older adults (older adults mean age = 69.9, SD = 3.6; older-old adults mean age = 83.5, SD = 3.3). The CFQ shows sufficient structural validity (CFA: *χ*^2^ (106, *N* = 449) = 260.46, *p* = 0.011, CFI = 0.985, SRMR = 0.036, RMSEA = 0.023) as well as internal consistency (Cronbach’s *α* = 0.93). On the other hand, the PRMQ also shows sufficient structural validity (CFA: *χ*^2^ (88, *N* = 464) = 186.14, *p* < 0.001, CFI = 0.959, SRMR = 0.035, RMSEA = 0.049) and internal consistency (Cronbach’s *α* = 0.84, for self-rated memory; *α* = 0.84, for self-rated prospective memory; and *α* = 0.79, for self-rated retrospective memory).

The Subjective Cognitive Decline Questionnaire (SCD-Q) is a 24-item scale that was validated in a cohort of 794 Spanish speakers to assess self-perceived change in several cognitive domains in older adults over the preceding 2 years [40]. The scale has a self and informant parts, each consisting of the same 24 items. The response options are dichotomous (yes/no) and the total score for each part of the questionnaire ranged from 0 to 24 points. The authors report sufficient internal consistency for the self (Cronbach’s *α* = 0.90) and informant parts (Cronbach’s *α* = 0.93). Structural validity of the SCD-Q was investigated in the original study as well as another included study conducted in Spain, both using EFA [40, 42], and therefore, both studies were judged to have “indeterminate” quality of structural validity.

The Cognitive Change Index (CCI) is a 20-item scale, developed and validated in a sample of 267 older adults in the USA [41]. It has a self and informant’s parts consisting of the same questions. The items are rated on a 5-point Likert scale ranging from 1 = no change or normal ability, 2 = minimal change or slight/occasional problem, 3 = some change or mild problem, 4 = clearly noticeable change or moderate problem, to 5 = much worse or severe problem. The authors report sufficient internal consistency (Cronbach *α*: self = 0.96, informant = 0.98). No structural validity analysis was reported. Therefore, internal consistency could not be rated or considered sufficient.

The Cognitive Dysfunction Questionnaire (CDQ) is a 20-item questionnaire that was validated in a sample of 794 older adults in Sweden [43]. The questionnaire aims to assess changes in cognitive function over the preceding 1 year. The items are rated on a 5-point Likert scale ranging from very seldom = 1, seldom =2, sometimes = 3, often = 4, and very often = 5. EFA was performed and the scale showed sufficient structural validity (the Kaiser-Meyer-Olkin measure of sampling adequacy = 0.95, Bartlett’s test of sphericity: v2 = 20,254, *p* < 0.001). Moreover, internal consistency was considered sufficient with a reported Cronbach’s *α* of 0.90. Convergent validity was evaluated against another measure of self-report cognitive decline, the PRMQ [50], which was of sufficient quality (*r* = 0.40, *p* < 0.001). The same questionnaire was evaluated by the same first author to assess the structural validity of a refined version that has 20 more items using CFA. The reported results indicate sufficient structural validity of the refined version of the CDQ (S-B *χ*^2^ = 558.5, df = 165, *p* < 0.000, RMSEA = 0.046 (CI; 0.042–0.050), SRMR = 0.057, CFI = 0.98).

The Subjective Memory Complaints Questionnaire (SMCQ) is a 14-item scale that assesses subjective memory decline [45]. The authors report developing and validating the questionnaire in a sample of 1651 older adults in South Korea (mean age 74.3, SD = 8.2). The response options are either yes or no. The highest possible total score on the SMCQ is 14 points (SMCQ-T): 4 points for the judgment of global memory (SMCQ-G) and 10 points for everyday memory (SMCQ-E). The authors demonstrated that the SMCQ has sufficient structural validity by performing CFA. The reported goodness-of-fit index (GFI), comparative fit index (CFI), Tucker-Lewis index (TLI), and root mean square error of approximation (RMSEA) indices for model-fitting were 0.961, 0.929, 0.940, and 0.54, respectively. The internal consistency of the SMCQ was good and was considered to be sufficient (Cronbach’s *α*: SMCQ-T = 0.864, SMCQ-G = 0.827, SMCQ-E = 0.694). The authors also report sufficient test-retest reliability of the SMCQ, SMCQ-G, and SMCQ-E (0.828, *p* = 0.001; 0.471, *p* = 0.03; and 0.836, *p* = 0.001, respectively).

## Discussion

We conducted a systematic review to evaluate the reported measurement properties of SCD PROMs that are used to assess and detect SCD in older adults in the context of AD. We identified and included 16 studies that developed and/or validated 17 self-report measures that assess SCD. We examined the reported development procedure and the internal structure of the measures and provided a comprehensive evaluation of their risk of bias and methodological quality. Our findings suggest that currently available SCD PROMs do not address important psychometric properties.

SCD is an emerging construct that is being considered as one of the earliest clinical symptoms of AD [7, 12]. Our findings suggest that several SCD PROMs show sufficient structural validity and internal consistency. However, despite this, we were not able to formulate a recommendation for which SCD measure is most adequate to use. This is due to several factors. First, none of the included studies conducted a content validity study. Content validity is the extent to which a measure truly captures the construct that it was developed to measure [21]. It is considered the most important, yet most challenging aspect of a PROM development and validation process [22]. It involves a thorough procedure of asking patients and professionals to ensure that all items included in a measure are relevant to the construct under evaluation, are well comprehended by both the target population and professionals (e.g., health professionals or researchers), and are comprehensive enough to include all important aspects of the construct [21, 22]. Because no content validity evaluation was available, and as recommended by the COSMIN guidelines, we could not evaluate the overall quality of the PROM nor provide an assessment of the confidence of collected evidence to present a recommended SCD PROM. Secondly, only one study reported the administration time of the developed PROM (the spatial navigation questionnaire, average time of administration = 5 min) [30]. This questionnaire, however, evaluates only subjective change in only one cognition domain. The majority of the included studies also failed to specify the mode (e.g., pen-and-paper versus electronic) and the proposed setting of administration (e.g., clinical setting versus at home). Without knowing how long each questionnaire takes or how it should be administered, it is difficult to infer the level of feasibility of the questionnaire at hand.

Some limitations of the present systematic review are worth mentioning. For inclusion in the systematic review, studies needed to have reported psychometric evaluation of questionnaires assessing SCD in older adults in the context of AD. This led to the exclusion of self-reported to questionnaires that were developed to assess the construct of SCD in general and were developed or validated in other populations. Indeed, valuable insights can be gathered from these questionnaires, some of which may be well-validated questionnaire that reliably assesses the same construct of interest [51]. However, including indirect evidence may introduce heterogeneity because participants in different studies would differ greatly in their sociodemographic and risk factors than those with or at risk of AD [51]. Moreover, our aim was to understand the current status of SCD self-reported questionnaire that are specifically designed to assess SCD related to AD. Therefore, the review team decided to only consider evidence that addressed and included populations with or at risk of AD and that validated questionnaires for the sole purpose of identifying SCD in the context of AD. In addition, all identified 17 PROMs were developed and validated in high-income settings (i.e., European countries the USA, South Korea). This sheds light on the importance of further cross-cultural validation of SCD measures, not only in other languages but also more importantly, in low-and-middle-income settings as well.

In conclusion, the results of this systematic review highlight the need for questionnaires that address important measurement properties in order to reliably assess SCD in older adults. A well-validated measure that is also feasible can aid in identifying older adults at risk of AD. Early identification would not only assist in including the right population for clinical trials, but also allow people the opportunity to be followed up closely and be offered meaningful intervention as early as possible.

## Supplementary Information

**Additional file 1: **Search strategy. **Table S1.** Reported results of the psychometric properties of the included SCD PROMs. **Table S2.** Summary of the assessment rating for the quality of measurement properties of the included SD PROMs. **Table S3.** Overview of excluded studies after full text revision, with citation.

## Data Availability

Data is available upon reasonable request.
