# Supplementary material for: Evaluating measurement properties of subjective cognitive decline self-reported outcome measures: a systematic review
Source: Syst Rev. 2022 Jul 18;11:144. doi: 10.1186/s13643-022-02018-y (PMC9290248; doi:10.1186/s13643-022-02018-y)
Supplement: Supplementary file 1 — Additional file 1: Search strategy. Table S1. Reported results of the psychometric properties of the included SCD PROMs. Table S2. Summary of the assessment rating for the quality of measurement properties of the included SD PROMs. Table S3. Overview of excluded studies after full text revision, with citation. [file 13643_2022_2018_MOESM1_ESM.docx]

**Appendix**

- **Search strategy**

1. **MEDLINE/PubMed search:**

Search date: 03.06.2020. Search results = 43

(subjective[All Fields] AND ("cognitive dysfunction"[MeSH Terms] OR ("cognitive"[All Fields] AND "dysfunction"[All Fields]) OR "cognitive dysfunction"[All Fields] OR ("cognitive"[All Fields] AND "impairment"[All Fields]) OR "cognitive impairment"[All Fields])) AND (preclinical[All Fields] AND ("alzheimer disease"[MeSH Terms] OR ("alzheimer"[All Fields] AND "disease"[All Fields]) OR "alzheimer disease"[All Fields] OR "alzheimer"[All Fields]) AND "cognitive dysfunction"[MeSH Terms] OR ("cognitive"[All Fields] AND "dysfunction"[All Fields]) OR "cognitive dysfunction"[All Fields] OR ("mild"[All Fields] AND "cognitive"[All Fields] AND "impairment"[All Fields]) OR "mild cognitive impairment"[All Fields] AND ("alzheimer disease"[MeSH Terms] OR ("alzheimer"[All Fields] AND "disease"[All Fields]) OR "alzheimer disease"[All Fields] OR ("alzheimer's"[All Fields] AND "disease"[All Fields]) OR "alzheimer's disease"[All Fields]) AND ("dementia"[MeSH Terms] OR "dementia"[All Fields])) AND (("self report"[MeSH Terms] OR ("self"[All Fields] AND "report"[All Fields]) OR "self report"[All Fields] OR ("self"[All Fields] AND "reported"[All Fields]) OR "self reported"[All Fields]) AND ("surveys and questionnaires"[MeSH Terms] OR ("surveys"[All Fields] AND "questionnaires"[All Fields]) OR "surveys and questionnaires"[All Fields] OR "questionnaire"[All Fields])) AND ((instrumentation[sh] OR methods[sh] OR "psychometrics"[MeSH] OR psychometr*[tiab] OR clinimetr*[tw] OR clinometr*[tw] OR "outcome assessment (health care)"[MeSH] OR "outcome assessment"[tiab] OR "outcome measure*"[tw] OR "observer variation"[MeSH] OR "observer variation"[tiab] OR "Health Status Indicators"[Mesh] OR "reproducibility of results"[MeSH] OR reproducib*[tiab] OR "discriminant analysis"[MeSH] OR reliab*[tiab] OR unreliab*[tiab] OR valid*[tiab] OR "coefficient of variation"[tiab] OR coefficient[tiab] OR homogeneity[tiab] OR homogeneous[tiab] OR "internal consistency"[tiab] OR (cronbach*[tiab] AND (alpha[tiab] OR alphas[tiab])) OR (item[tiab] AND (correlation*[tiab] OR selection*[tiab] OR reduction*[tiab])) OR agreement[tw] OR precision[tw] OR imprecision[tw] OR "precise values"[tw] OR test-retest[tiab] OR (test[tiab] AND retest[tiab]) OR (reliab*[tiab] AND (test[tiab] OR retest[tiab])) OR stability[tiab] OR interrater[tiab] OR inter-rater[tiab] OR intrarater[tiab] OR intra-rater[tiab] OR intertester[tiab] OR inter-tester[tiab] OR intratester[tiab] OR intra-tester[tiab] OR interobserver[tiab] OR inter-observer[tiab] OR intraobserver[tiab] OR intra-observer[tiab] OR intertechnician[tiab] OR inter-technician[tiab] OR intratechnician[tiab] OR intra-technician[tiab] OR interexaminer[tiab] OR inter-examiner[tiab] OR intraexaminer[tiab] OR intra-examiner[tiab] OR interassay[tiab] OR inter-assay[tiab] OR intraassay[tiab] OR intra-assay[tiab] OR interindividual[tiab] OR inter-individual[tiab] OR intraindividual[tiab] OR intra-individual[tiab] OR interparticipant[tiab] OR inter-participant[tiab] OR intraparticipant[tiab] OR intra-participant[tiab] OR kappa[tiab] OR kappa's[tiab] OR kappas[tiab] OR repeatab*[tw] OR ((replicab*[tw] OR repeated[tw]) AND (measure[tw] OR measures[tw] OR findings[tw] OR result[tw] OR results[tw] OR test[tw] OR tests[tw])) OR generaliza*[tiab] OR generalisa*[tiab] OR concordance[tiab] OR (intraclass[tiab] AND correlation*[tiab]) OR discriminative[tiab] OR "known group"[tiab] OR "factor analysis"[tiab] OR "factor analyses"[tiab] OR "factor structure"[tiab] OR "factor structures"[tiab] OR dimension*[tiab] OR subscale*[tiab] OR (multitrait[tiab] AND scaling[tiab] AND (analysis[tiab] OR analyses[tiab])) OR "item discriminant"[tiab] OR "interscale correlation*"[tiab] OR error[tiab] OR errors[tiab] OR "individual variability"[tiab] OR "interval variability"[tiab] OR "rate variability"[tiab] OR (variability[tiab] AND (analysis[tiab] OR values[tiab])) OR (uncertainty[tiab] AND (measurement[tiab] OR measuring[tiab])) OR "standard error of measurement"[tiab] OR sensitiv*[tiab] OR responsive*[tiab] OR (limit[tiab] AND detection[tiab]) OR "minimal detectable concentration"[tiab] OR interpretab*[tiab] OR ((minimal[tiab] OR minimally[tiab] OR clinical[tiab] OR clinically[tiab]) AND (important[tiab] OR significant[tiab] OR detectable[tiab]) AND (change[tiab] OR difference[tiab])) OR (small*[tiab] AND (real[tiab] OR detectable[tiab]) AND (change[tiab] OR difference[tiab])) OR "meaningful change"[tiab] OR "ceiling effect"[tiab] OR "floor effect"[tiab] OR "Item response model"[tiab] OR IRT[tiab] OR Rasch[tiab] OR "Differential item functioning"[tiab] OR DIF[tiab] OR "computer adaptive testing"[tiab] OR "item bank"[tiab] OR "cross-cultural equivalence"[tiab])) AND (("1982/01/01"[PDAT] : "2020/12/31"[PDAT]) AND English[lang])

1. **Embase:**

Search date: 27.05.2020. Search results = 212

'subjective' AND ('cognitive defect'/exp OR 'cognitive defect') AND ('psychometry'/exp OR 'psychometry') AND [english]/lim AND [embase]/lim AND [1982-2020]/py

1. **PsycINFO:**

Search date: 27.05.2020. Search results = 47

(((((((DE "Cognitive Impairment") OR (DE "Dementia")) OR (DE "Memory Disorders")) OR (DE "Neurocognitive Disorders")) AND (DE "Subjectivity")) AND (DE "Patient Reported Outcome Measures" OR DE "Self-Report")) AND (DE "Psychometrics" OR DE "Measurement" OR DE "Classical Test Theory" OR DE "Consistency (Measurement)" OR DE "Error of Measurement" OR DE "Factor Analysis" OR DE "Item Analysis (Test)" OR DE "Item Response Theory" OR DE "Measurement Invariance" OR DE "Measurement Models" OR DE "Multivariate Analysis" OR DE "Test Construction" OR DE "Test Reliability" OR DE "Test Sensitivity" OR DE "Test Specificity" OR DE "Test Validity" OR DE "Variability Measurement" OR DE "Conjoint Measurement" OR DE "Experimental Design" OR DE "Statistical Analysis" OR DE "Test Interpretation" OR DE "Test Revision" OR DE "Testing")

1. **Pingree database**

Search date: 29.05.2020. Search results = 9

subjective cognitive decline

1. **Open Access Theses and Dissertations**

Search date: 29.05.2020. Search results = 0

subjective cognitive decline AND alzheimer's disease AND self report AND psychometrics

1. **WorldCat**

Search date: 10.06.2020. Search results = 6

'[kw:subjective cognitive decline kw:self report kw:psychometry kw:Alzheimer](https://www.worldcat.org/search?q=kw%3Asubjective+cognitive+decline+kw%3Aself+report+kw%3Apsychometry+kw%3AAlzheimer&qt=facetNavigation&dblist=638)' > '[1982..2020](https://www.worldcat.org/search?q=kw%3Asubjective+cognitive+decline+kw%3Aself+report+kw%3Apsychometry+kw%3AAlzheimer&qt=facetNavigation&fq=yr%3A1982..2020&dblist=638)' >

1. **Web of Science**

Search date: 15.06.2020. Search results = 1

(ALL=Subjective cognitive decline  AND ALL=Alzheimer  AND ALL=self-report  AND ALL=psychometr*)  AND LANGUAGE: (English) Timespan: 1982-2020. Indexes: SCI-EXPANDED, SSCI, A&HCI, ESCI.

1. **Scopus**

Search date: 29.05.2020. Search results = 46

subjective  AND  cognitive  AND decline  AND  alzheimer's  AND disease  AND self-report  AND  questionnaire  AND  psychometry  AND  ( LIMIT-TO ( LANGUAGE ,  "English" ) )

- **Supplementary tables**

*Table S1: Reported results of the psychometric properties of the included SCD PROMs*

| *Psychometric properties* | | | | | |
| --- | --- | --- | --- | --- | --- |
| *First author (Year)* | ***Structural validity*** | ***Internal consistency (95%CI)*** | ***Test-retest reliability (95% CI)*** | ***Cross-cultural validity*** | ***Convergent validity*** |
| *Allison et al. (2019)* | EFA - Uni, RMSEA = 0.76; ratio = 10.150 | Subject: α = .0965 (0.953–0.974) Informant: α = 0.957 (0.942–0.970) | ICC:  Subject = 0.838 (0.743–0.900) Informant = 0.723 (0.552–0.835) |  |  |
| *Avila-Villanueva et al. (2016)* | EFA | NA (mentioned but no alpha reported) |  | Translation |  |
| *Chipi et al. (2018)* |  | Subject: α = 0.77 (0.72–0.83) Informant-relatives: 0.77 (0.70–0.85) Informant-volunteers:0.72 (0.66-0.78) |  | Translation |  |
| *Crook et al. (1992)* |  | α = 0.57 | Unclear (= 0.67) |  | r = .41, p-value < 0.001 |
| *Crowe et al. (2006)* |  | α = 0.81 |  |  |  |
| *Crowe et al. (2006)* |  | α = 0.92 |  |  |  |
| *Gifford et al. (2015)* | EFA, CFA | θ score range: Cognitively healthy participants = 20.12 - 0.90  participants with MCI = 0.34 - 0.83 |  |  |  |
| *Gilewski et al. (1990)* | EFA, CFA | α = 0.94, 0.94, 0.89, and 0.83 for each factor |  |  |  |
| *La Joie et al. (2016)* | EFA |  |  | Translation |  |
| *Lubitz et al. (2018)* | CFA: χ² (112) = 318.29, p < .001, CFI = .97, RMSEA = .05 (95% CI: .04–.06) | α = 0.87 |  |  |  |
| *Papaliagkas et al. (2017)* | CFA: χ² (106, N = 449) = 260.46, p = .011, CFI = .985, SRMR = .036, RMSEA = .023 | α = 0.93 |  | Translation |  |
| *Papaliagkas et al. (2017)* | CFA: χ² (88, N = 464) = 186.14, p < .001, CFI = .959, SRMR = .035, RMSEA = .049 | Self-Rated Memory α = .84  Self-Rated Prospective Memory α = .84  Self-Rated Retrospective Memory α = .79  (no CI was reported) |  | Translation |  |
| *Rami et al. (2014)* | EFA: KMO value = 0.94; Bartlett’s Test of Sphericity < 0.001 | Subject α = 0.90  Informant α = 0.93 |  |  |  |
| *Rattanabannakit et al. (2016)* |  | Subject α = 0.96 Informant α = 0.98 |  |  |  |
| *Valech et al. (2018)* | EFA: KMO = 0.824  significant Bartlett’s Test = 880.43; p < 0.0001) |  |  |  |  |
| *Vestergren et al. (2011)* | EFA: KMO = 0.95  Bartlett’s test of sphericity: v2 = 20254, p < 0.001 | 0.9 |  |  | r = 0.40, p < 0.001 |
| *Vestergren et al. (2012)* | CFA: S-Bχ² = 558.5, df = 165, p < 0.000, RMSEA = 0.046 (CI; 0.042–0.050), SRMR = 0.057, CFI = 0.98 |  |  |  |  |
| *Youn et al. (2009)* | GFI = 0.961, CFI = 0.929, TLI = 0.940, RMSEA = 0.54 | SMCQ-T α = 0.864  SMCQ-G α = 0.827  SMCQ-E α = 0.694 | SMCQ-T: 0.828, p = 0.001  SMCQ-G: 0.471, p = 0.03  SMCQ-E: 0.836, p = 0.001 |  |  |

Abbreviations. α, Cronbach’s alpha; CFI, comparative fit index; CFA, Confirmatory factor Analysis; CI, confidence interval; df, degrees of freedom; EFA, Exploratory factor Analysis; GFI, goodness-of-fit index; ICC, Inter-Class Coefficient; KMO, Kaiser-Meyer-Olkin; r, Pearson’s correlation coefficient, RMSEA, root mean square error of approximation; SRMR, Standardized Root Mean Square Residual; TLI, Tucker-Lewis index.

*Table S2: Summary of the assessment rating for the quality of measurement properties of the included SD PROMs*

|  | *Psychometric properties* | | | | |
| --- | --- | --- | --- | --- | --- |
| *First author (Year)* | ***Structural validity*** | ***Internal consistency: α (CI)*** | ***Test-retest reliability: ICC (CI)*** | ***Cross-cultural validity*** | ***Convergent validity*** |
| *Allison et al. (2019)* | ? | + | + |  |  |
| *Avila-Villanueva et al. (2016)* | ? | ? |  | + |  |
| *Chipi et al. (2018)* |  | + |  | + |  |
| *Crook et al. (1992)* |  | - | ? |  | + |
| *Crowe et al. (2006)* |  | + |  |  |  |
| *Crowe et al. (2006)* |  | + |  |  |  |
| *Gifford et al. (2015)* | + | + |  |  |  |
| *Gilewski et al. (1990)* | + | + | - |  |  |
| *La Joie et al. (2016)* | + |  |  | + |  |
| *Lubitz et al. (2018)* | + | + |  |  |  |
| *Papaliagkas et al. (2017)* | + | + |  | + |  |
| *Papaliagkas et al. (2017)* | + | + |  | + |  |
| *Rami et al. (2014)* | ? | + |  |  |  |
| *Rattanabannakit et al. (2016)* | - | ? |  |  |  |
| *Valech et al. (2018)* | ? |  |  |  |  |
| *Vestergren et al. (2011)* | + | + |  |  | + |
| *Vestergren et al. (2012)* | + |  |  |  |  |
| *Youn et al. (2009)* | + | + |  |  |  |

Note. Each result per psychometric property is rated as either sufficient (+), insufficient (–), or indeterminate (?)

*Table S3: Overview of excluded studies after full text revision, with citation*

| *Rayyan ID* | *Author* | *Year* | *Reason* | *Citation* |
| --- | --- | --- | --- | --- |
| *82877490* | ***Anderson*** | 2018 | Wrong publication type | Anderson Deborah. Cognitive assessment. In: The elements of psychological case report writing in Australia. Routledge/Taylor & Francis Group; 2018. p. 52–9. |
| *82877636* | ***Balogh*** | 2020 | Measure is not a SCD PROM | Balogh N, Åstrand R, Wallin A, Rolstad S. The five‐items memory screen‐extended variant: A tool for assessing memory. Acta Neurol Scand [Internet]. 2020 Feb [cited 2022 Jun 23];141(2):162–7. Available from: <https://onlinelibrary.wiley.com/doi/10.1111/ane.13188> |
| *82877517* | ***Bhushan*** | 2018 | Validation of SCD PROM not the aim of the study | Bhushan A, Fondell E, Ascherio A, Yuan C, Grodstein F, Willett W. Adherence to Mediterranean diet and subjective cognitive function in men. Eur J Epidemiol [Internet]. 2018 Feb [cited 2022 Jun 23];33(2):223–34. Available from: <http://link.springer.com/10.1007/s10654-017-0330-3> |
| *82877504* | ***Buelow*** | 2014 | Validation of SCD PROM not the aim of the study | Buelow MT, Tremont G, Frakey LL, Grace J, Ott BR. Utility of the Cognitive Difficulties Scale and Association With Objective Test Performance. Am J Alzheimers Dis Other Demen [Internet]. 2014 Dec [cited 2022 Jun 23];29(8):755–61. Available from: <http://journals.sagepub.com/doi/10.1177/1533317514539032> |
| *82877677* | ***Caselli*** | 2014 | Validation of SCD PROM not the aim of the study | Caselli RJ, Chen K, Locke DEC, Lee W, Roontiva A, Bandy D, et al. Subjective cognitive decline: Self and informant comparisons. Alzheimer’s &amp; Dementia [Internet]. 2014 Jan [cited 2022 Jun 23];10(1):93–8. Available from: <https://onlinelibrary.wiley.com/doi/10.1016/j.jalz.2013.01.003> |
| *82877468* | ***Chipi*** | 2019 | Validation of SCD PROM not the aim of the study | Chipi E, Montanucci C, Eusebi P, D’Andrea K, Biscetti L, Calabresi P, et al. The Italian version of Cognitive Function Instrument (CFI) for tracking changes in healthy elderly: results at 1-year follow-up. Neurol Sci [Internet]. 2019 Oct [cited 2022 Jun 23];40(10):2147–53. Available from: <http://link.springer.com/10.1007/s10072-019-03960-x> |
| *82877503* | ***Clément*** | 2008 | Validation of SCD PROM not the aim of the study | Clément F, Belleville S, Gauthier S. Cognitive complaint in mild cognitive impairment and Alzheimer’s disease. J Inter Neuropsych Soc [Internet]. 2008 Mar [cited 2022 Jun 23];14(02). Available from: <http://www.journals.cambridge.org/abstract_S1355617708080260> |
| *82877538* | ***Davoudkhani*** | 2019 | Wrong construct | Davoudkhani M, Kormi-Nouri R, Norouzi Javidan A, Sharifi F, Younesi F, Zendehbad AS, et al. The Validity and Reliability of a Persian Version of the Brief Community Screening Instrument for Dementia in the Elderly Patients with Dementia in Iran. Arch Neurosci [Internet]. 2019 Jul 28 [cited 2022 Jun 23];6(3). Available from: <https://brief.land/ans/articles/92611.html> |
| *82877486* | ***De Souza*** | 2011 | Measure is not a SCD PROM | Souza AS de, Oliveira-Souza R de, Moll J, Tovar-Moll F, Andreiuolo PA, Bottino CMC. Contribution of 1H Spectroscopy to a Brief Cognitive-Functional Test Battery for the Diagnosis of Mild Alzheimer’s Disease. DEM [Internet]. 2011 [cited 2022 Jun 23];32(5):351–61. Available from: <https://www.karger.com/Article/FullText/334656> |
| *82877496* | ***Dowling*** | 2016 | Measure is not a SCD PROM | Dowling NM, Bolt DM, Deng S. An approach for estimating item sensitivity to within-person change over time: An illustration using the Alzheimer’s Disease Assessment Scale–Cognitive subscale (ADAS-Cog). Psychological Assessment [Internet]. 2016 Dec [cited 2022 Jun 23];28(12):1576–85. Available from: <http://doi.apa.org/getdoi.cfm?doi=10.1037/pas0000285> |
| *82877505* | ***Edmonds*** | 2014 | Validation of SCD PROM not the aim of the study | Edmonds EC, Delano-Wood L, Galasko DR, Salmon DP, Bondi MW. Subjective Cognitive Complaints Contribute to Misdiagnosis of Mild Cognitive Impairment. J Int Neuropsychol Soc [Internet]. 2014 Sep [cited 2022 Jun 23];20(8):836–47. Available from: <https://www.cambridge.org/core/product/identifier/S135561771400068X/type/journal_article> |
| *82877522* | ***Edmonds*** | 2018 | Validation of SCD PROM not the aim of the study | Edmonds EC, Weigand AJ, Thomas KR, Eppig J, Delano-Wood L, Galasko DR, et al. Increasing Inaccuracy of Self-Reported Subjective Cognitive Complaints Over 24 Months in Empirically Derived Subtypes of Mild Cognitive Impairment. J Int Neuropsychol Soc [Internet]. 2018 Sep [cited 2022 Jun 23];24(8):842–53. Available from: <https://www.cambridge.org/core/product/identifier/S1355617718000486/type/journal_article> |
| *82877467* | ***Farias*** | 2008 | Wrong population | Farias ST, Mungas D, Reed BR, Cahn-Weiner D, Jagust W, Baynes K, et al. The measurement of everyday cognition (ECog): Scale development and psychometric properties. Neuropsychology [Internet]. 2008 [cited 2022 Jun 23];22(4):531–44. Available from: <http://doi.apa.org/getdoi.cfm?doi=10.1037/0894-4105.22.4.531> |
| *82877495* | ***Fox*** | 2011 | Validation of SCD PROM not the aim of the study | Fox C, Richardson K, Maidment ID, Savva GM, Matthews FE, Smithard D, et al. Anticholinergic Medication Use and Cognitive Impairment in the Older Population: The Medical Research Council Cognitive Function and Ageing Study: ANTICHOLINERGIC ACTIVITY AND COGNITION. Journal of the American Geriatrics Society [Internet]. 2011 Aug [cited 2022 Jun 23];59(8):1477–83. Available from: <https://onlinelibrary.wiley.com/doi/10.1111/j.1532-5415.2011.03491.x> |
| *82877491* | ***Garica-Alberca*** | 2011 | Validation of SCD PROM not the aim of the study | García-Alberca JM, Lara JP, Berthier ML, Cruz B, Barbancho MÁ, Green C, et al. Can impairment in memory, language and executive functions predict neuropsychiatric symptoms in Alzheimer’s disease (AD)? Findings from a cross-sectional study. Archives of Gerontology and Geriatrics [Internet]. 2011 May [cited 2022 Jun 23];52(3):264–9. Available from: <https://linkinghub.elsevier.com/retrieve/pii/S0167494310001299> |
| *82877484* | ***Gerstorf*** | 2008 | Wrong population | Gerstorf D, Siedlecki KL, Tucker-Drob EM, Salthouse TA. Executive Dysfunctions Across Adulthood: Measurement Properties and Correlates of the DEX Self-Report Questionnaire. Aging, Neuropsychology, and Cognition [Internet]. 2008 Jul 4 [cited 2022 Jun 23];15(4):424–45. Available from: <http://www.tandfonline.com/doi/abs/10.1080/13825580701640374> |
| *82877457* | ***Girtler*** | 2012 | Measure is not a SCD PROM | Girtler N, Brugnolo A, Campus C, De Carli F, Famà F, Ghio L, et al. The short cognitive evaluation battery in cognitive disorders of the elderly--Italian version. Dement Geriatr Cogn Disord. 2012;33(4):255–65. |
| *82877519* | ***Hsu*** | 2019 | Validation of SCD PROM not the aim of the study | Hsu YH, Huang CF, Huang WH, Deng JF, Tu MC. Microstructural Correlates and Laterality Effect of Prospective Memory in Non-Demented Adults with Memory Complaints. Dement Geriatr Cogn Disord [Internet]. 2019 [cited 2022 Jun 23];47(4–6):375–84. Available from: <https://www.karger.com/Article/FullText/501366> |
| *NA* | ***Jorm*** | 1994 | Validation of SCD PROM not the aim of the study | Jorm AF, Christensen H, Henderson AS, Korten AE, Mackinnon AJ, Scott R. Complaints of cognitive decline in the elderly: a comparison of reports by subjects and informants in a community survey. Psychol Med [Internet]. 1994 May [cited 2022 Jun 23];24(2):365–74. Available from: <https://www.cambridge.org/core/product/identifier/S0033291700027343/type/journal_article> |
| *82877706* | ***Jungwirth*** | 2009 | Validation of SCD PROM not the aim of the study | Jungwirth S, Zehetmayer S, Bauer P, Weissgram S, Tragl KH, Fischer P. Screening for Alzheimer’s dementia at age 78 with short psychometric instruments. IPG [Internet]. 2009 Jun [cited 2022 Jun 23];21(03):548. Available from: <http://www.journals.cambridge.org/abstract_S1041610209008904> |
| *82877530* | ***Jutten*** | 2017 | Wrong construct | Jutten RJ, Peeters CFW, Leijdesdorff SMJ, Visser PJ, Maier AB, Terwee CB, et al. Detecting functional decline from normal aging to dementia: Development and validation of a short version of the Amsterdam IADL Questionnaire. Alzheimer’s &amp; Dementia: Diagnosis, Assessment &amp; Disease Monitoring [Internet]. 2017 Jan [cited 2022 Jun 23];8(1):26–35. Available from: <https://onlinelibrary.wiley.com/doi/abs/10.1016/j.dadm.2017.03.002> |
| *82877516* | ***Kielb*** | 2017 | Validation of SCD PROM not the aim of the study | Kielb S, Rogalski E, Weintraub S, Rademaker A. Objective features of subjective cognitive decline in a United States national database. Alzheimer’s &amp; Dementia [Internet]. 2017 Dec [cited 2022 Jun 23];13(12):1337–44. Available from: <https://onlinelibrary.wiley.com/doi/10.1016/j.jalz.2017.04.008> |
| *NA* | ***Kogler*** | 2016 | Study not reported in English | Kogler, S. Subjektive Gedächtniseinschätzung Bei Patientinnen Mit Mild Cognitive Impairment, Alzheimerkrankheit Und Parkinsonkrankheit. Vienna: University of Vienna; 2013. Available from [https://phaidra.univie.ac.at/download/o:1304429] |
| *82877537* | ***Kuhn*** | 2019 | Validation of SCD PROM not the aim of the study | Kuhn E, Moulinet I, Perrotin A, La Joie R, Landeau B, Tomadesso C, et al. Cross-sectional and longitudinal characterization of SCD patients recruited from the community versus from a memory clinic: subjective cognitive decline, psychoaffective factors, cognitive performances, and atrophy progression over time. Alz Res Therapy [Internet]. 2019 Dec [cited 2022 Jun 23];11(1):61. Available from: <https://alzres.biomedcentral.com/articles/10.1186/s13195-019-0514-z> |
| *82877587* | ***Kurt*** | 2011 | Validation of SCD PROM not the aim of the study | Kurt P, Yener G, Oguz M. Impaired digit span can predict further cognitive decline in older people with subjective memory complaint: A preliminary result. Aging & Mental Health [Internet]. 2011 Apr 1 [cited 2022 Jun 23];15(3):364–9. Available from: <https://www.tandfonline.com/doi/full/10.1080/13607863.2010.536133> |
| *82877555* | ***Lowe*** | 2015 | Measure is not a SCD PROM | Lowe DA, Balsis S, Benge JF, Doody RS. Adding delayed recall to the ADAS-cog improves measurement precision in mild Alzheimer’s disease: Implications for predicting instrumental activities of daily living. Psychological Assessment [Internet]. 2015 Dec [cited 2022 Jun 23];27(4):1234–40. Available from: <http://doi.apa.org/getdoi.cfm?doi=10.1037/pas0000133> |
| *82877458* | ***Mackinnon*** | 2001 | Measure is not a SCD PROM | Mackinnon A, Mulligan R. The Psychogeriatric Assessment Scales (PAS): psychometric properties in French and German speaking populations. Int J Geriat Psychiatry [Internet]. 2001 Sep [cited 2022 Jun 23];16(9):892–9. Available from: <https://onlinelibrary.wiley.com/doi/10.1002/gps.442> |
| *82877624* | ***Marri*** | 2001 | Validation of SCD PROM not the aim of the study | Marri L, Modugno M, Iacono S, Renzetti C, De Vreese LP, Neri M. Metamemory and self-perceived health in mild cognitive impairment. Arch Gerontol Geriatr Suppl. 2001;7:235–44. |
| *82877498* | ***Martínez-Martín*** | 2012 | Wrong construct | Martínez-Martín P, Osa-Ruiz E, Gómez-Conesa A, Olazarán J. A Rating Scale for Gait Evaluation in Cognitive Deterioration (RSGE-CD): Validation Study. JAD [Internet]. 2012 Aug 22 [cited 2022 Jun 23];31(3):543–53. Available from: <https://www.medra.org/servlet/aliasResolver?alias=iospress&doi=10.3233/JAD-2012-120271> |
| *82877509* | ***Matsuzawa*** | 2012 | Validation of SCD PROM not the aim of the study | Matsuzawa T, Takata T, Yokono K, Ueda H, Moriwaki K, Kamae I, et al. A Warning Index Used in Prescreening for Alzheimer’s Disease, Based on Self-Reported Cognitive Deficits and Vascular Risk Factors for Dementia in Elderly Patients with Type 2 Diabetes. International Journal of Alzheimer’s Disease [Internet]. 2012 [cited 2022 Jun 23];2012:1–8. Available from: <http://www.hindawi.com/journals/ijad/2012/124215/> |
| *82877472* | ***Mungas*** | 2003 | Validation of SCD PROM not the aim of the study | Mungas D, Reed BR, Kramer JH. Psychometrically matched measures of global cognition, memory, and executive function for assesment of cognitive decline in older persons. Neuropsychology [Internet]. 2003 [cited 2022 Jun 23];17(3):380–92. Available from: <http://doi.apa.org/getdoi.cfm?doi=10.1037/0894-4105.17.3.380> |
| *82877684* | ***Özel Kizil*** | 2013 | Study not reported in English | Özel Kızıl ET, Duman B, Altıntaş Ö, Kırıcı S, Baştuğ G, Baran Z, et al. Investigation of the Psychometric Properties of the Turkish Form of Subjective Memory Complaints Questionnaire. 2013 [cited 2022 Jun 23]; Available from: <http://earsiv.cankaya.edu.tr:8080/xmlui/handle/20.500.12416/2841> |
| *82877662* | ***Pendlebury*** | 2015 | Measure is not a SCD PROM | Pendlebury ST, Klaus SP, Mather M, de Brito M, Wharton RM. Routine cognitive screening in older patients admitted to acute medicine: abbreviated mental test score (AMTS) and subjective memory complaint versus Montreal Cognitive Assessment and IQCODE. Age Ageing [Internet]. 2015 Nov [cited 2022 Jun 23];44(6):1000–5. Available from: <https://academic.oup.com/ageing/article-lookup/doi/10.1093/ageing/afv134> |
| *82877667* | ***Pietrzak*** | 2015 | Validation of SCD PROM not the aim of the study | Pietrzak RH, Lim YY, Ames D, Harrington K, Restrepo C, Martins RN, et al. Trajectories of memory decline in preclinical Alzheimer’s disease: results from the Australian Imaging, Biomarkers and Lifestyle Flagship Study of Ageing. Neurobiology of Aging [Internet]. 2015 Mar [cited 2022 Jun 23];36(3):1231–8. Available from: <https://linkinghub.elsevier.com/retrieve/pii/S0197458014008318> |
| *82877514* | ***Polcher*** | 2017 | Validation of SCD PROM not the aim of the study | Polcher A, Frommann I, Koppara A, Wolfsgruber S, Jessen F, Wagner M. Face-Name Associative Recognition Deficits in Subjective Cognitive Decline and Mild Cognitive Impairment. JAD [Internet]. 2017 Feb 3 [cited 2022 Jun 23];56(3):1185–96. Available from: https://www.medra.org/servlet/aliasResolver?alias=iospress&doi=10.3233/JAD-160637 |
| *82877648* | ***Pons*** | 2018 | Validation of SCD PROM not the aim of the study | Pons A, LaMonica HM, Mowszowski L, Köhler S, Deckers K, Naismith SL. Utility of the LIBRA Index in Relation to Cognitive Functioning in a Clinical Health Seeking Sample. Hornberger M, editor. JAD [Internet]. 2018 Feb 6 [cited 2022 Jun 23];62(1):373–84. Available from: <https://www.medra.org/servlet/aliasResolver?alias=iospress&doi=10.3233/JAD-170731> |
| *82877536* | ***Poptsi*** | 2019 | Validation of SCD PROM not the aim of the study | Poptsi E, Moraitou D, Eleftheriou M, Kounti-Zafeiropoulou F, Papasozomenou C, Agogiatou C, et al. Normative Data for the Montreal Cognitive Assessment in Greek Older Adults With Subjective Cognitive Decline, Mild Cognitive Impairment and Dementia. J Geriatr Psychiatry Neurol [Internet]. 2019 Sep [cited 2022 Jun 23];32(5):265–74. Available from: <http://journals.sagepub.com/doi/10.1177/0891988719853046> |
| *82877511* | ***Rabin*** | 2015 | Validation of SCD PROM not the aim of the study | Rabin LA, Smart CM, Crane PK, Amariglio RE, Berman LM, Boada M, et al. Subjective Cognitive Decline in Older Adults: An Overview of Self-Report Measures Used Across 19 International Research Studies. Tales A, Jessen F, Butler C, Wilcock G, Phillips J, Bayer T, editors. JAD [Internet]. 2015 Sep 24 [cited 2022 Jun 23];48(s1):S63–86. Available from: <https://www.medra.org/servlet/aliasResolver?alias=iospress&doi=10.3233/JAD-150154> |
| *NA* | ***Rabin*** | 2007 | Validation of SCD PROM not the aim of the study | Rabin LA, Saykin AJ, Wishart HA, Nutter‐Upham KE, Flashman LA, Pare N, et al. The Memory and Aging Telephone Screen: Development and preliminary validation. Alzheimer’s &amp; Dementia [Internet]. 2007 Apr [cited 2022 Jun 23];3(2):109–21. Available from: <https://onlinelibrary.wiley.com/doi/10.1016/j.jalz.2007.02.002> |
| *82877521* | ***Rahman-filipiak*** | 2018 | Validation of SCD PROM not the aim of the study | Rahman-Filipiak AM, Giordani B, Heidebrink J, Bhaumik A, Hampstead BM. Self- and Informant-Reported Memory Complaints: Frequency and Severity in Cognitively Intact Individuals and those with Mild Cognitive Impairment and Neurodegenerative Dementias. Han D, editor. JAD [Internet]. 2018 Sep 11 [cited 2022 Jun 23];65(3):1011–27. Available from: <https://www.medra.org/servlet/aliasResolver?alias=iospress&doi=10.3233/JAD-180083> |
| *82877704* | ***Rami*** | 2010 | Measure is not a SCD PROM | Rami L, Bosch B, Sanchez-Valle R, Molinuevo JL. The memory alteration test (M@T) discriminates between subjective memory complaints, mild cognitive impairment and Alzheimer’s disease. Archives of Gerontology and Geriatrics [Internet]. 2010 Mar [cited 2022 Jun 23];50(2):171–4. Available from: <https://linkinghub.elsevier.com/retrieve/pii/S0167494309000764> |
| *82877602* | ***Reisberg*** | 2008 | Validation of SCD PROM not the aim of the study | Reisberg B, Gauthier S. Current evidence for subjective cognitive impairment (SCI) as the pre-mild cognitive impairment (MCI) stage of subsequently manifest Alzheimer’s disease. Int Psychogeriatr [Internet]. 2008 Feb [cited 2022 Jun 23];20(1):1–16. Available from: <https://www.cambridge.org/core/product/identifier/S1041610207006412/type/journal_article> |
| *82877459* | ***Salthouse*** | 2010 | Validation of SCD PROM not the aim of the study | Salthouse TA. The paradox of cognitive change. Journal of Clinical and Experimental Neuropsychology [Internet]. 2010 Jul 7 [cited 2022 Jun 23];32(6):622–9. Available from: <https://www.tandfonline.com/doi/full/10.1080/13803390903401310> |
| *82877525* | ***Silva*** | 2016 | Validation of SCD PROM not the aim of the study | Silva MR, Moser D, Pflüger M, Pusswald G, Stögmann E, Dal-Bianco P, et al. Self-reported and informant-reported memory functioning and awareness in patients with mild cognitive impairment and Alzheimer´s disease. Neuropsychiatr [Internet]. 2016 Jun [cited 2022 Jun 23];30(2):103–12. Available from: <http://link.springer.com/10.1007/s40211-016-0185-y> |
| *82877460* | ***Silva-filho*** | 2007 | Measure is not a SCD PROM | Silva-Filho JH, Pasian SR, do Vale F de AC. Typical performance of elderlypatients with Alzheimer disease on the Wisconsin Card Sorting Test (WCST). Dement Neuropsychol [Internet]. 2007 [cited 2022 Jun 23];1(2):181–9. Available from: <https://www.ncbi.nlm.nih.gov/pmc/articles/PMC5619567/> |
| *82877506* | ***Smart*** | 2014 | Measure is not a SCD PROM | Smart CM, Segalowitz SJ, Mulligan BP, MacDonald SWS. Attention capacity and self-report of subjective cognitive decline: A P3 ERP study. Biological Psychology [Internet]. 2014 Dec [cited 2022 Jun 23];103:144–51. Available from: <https://linkinghub.elsevier.com/retrieve/pii/S0301051114002014> |
| *82877469* | ***Sobral*** | 2015 | Wrong construct | Sobral M, Pestana MH, Paúl C. The impact of cognitive reserve on neuropsychological and functional abilities in Alzheimer’s disease patients. Psychology & Neuroscience [Internet]. 2015 [cited 2022 Jun 23];8(1):39–55. Available from: <http://doi.apa.org/getdoi.cfm?doi=10.1037/h0101022> |
| *82877665* | ***Sousa*** | 2015 | Measure is not a SCD PROM | Sousa M, Pereira A, Costa R, Rami L. Initial phase of adaptation of Memory Alteration Test (M@T) in a Portuguese sample. Archives of Gerontology and Geriatrics [Internet]. 2015 Jul [cited 2022 Jun 23];61(1):103–8. Available from: <https://linkinghub.elsevier.com/retrieve/pii/S0167494315000473> |
| *82877735* | ***Stites*** | 2017 | Wrong construct | Stites SD, Karlawish J, Harkins K, Rubright JD, Wolk D. Awareness of Mild Cognitive Impairment and Mild Alzheimer’s Disease Dementia Diagnoses Associated With Lower Self-Ratings of Quality of Life in Older Adults. The Journals of Gerontology: Series B [Internet]. 2017 Oct 1 [cited 2022 Jun 23];72(6):974–85. Available from: <https://academic.oup.com/psychsocgerontology/article/72/6/974/4004839> |
| *82877733* | ***Stites*** | 2018 | Validation of SCD PROM not the aim of the study | Stites SD, Harkins K, Rubright JD, Karlawish J. Relationships Between Cognitive Complaints and Quality of Life in Older Adults With Mild Cognitive Impairment, Mild Alzheimer Disease Dementia, and Normal Cognition. Alzheimer Dis Assoc Disord. 2018 Dec;32(4):276–83. |
| *82877734* | ***Szepietowska*** | 2018 | Validation of SCD PROM not the aim of the study | Szepietowska EM, Kuzaka A, Provincial Specialist Hospital, Biała Podlaska, Poland; Faculty of Tourism and Health, Józef Piłsudski University of Physical Education in Warsaw, Branch in Biała Podlaska, Biała Podlaska, Poland. Subjective executive difficulties – a study using the Dysexecutive Questionnaire. PSYCHIATR PSYCHOL KLIN [Internet]. 2018 Mar 29 [cited 2022 Jun 23];18(1):25–34. Available from: <http://www.psychiatria.com.pl/index.php/wydawnictwa/2018-vol-18-no-1/subjective-executive-difficulties-a-study-using-the-dysexecutive-questionnaire?aid=729> |
| *82877721* | ***Teresi*** | 2000 | Measure is not a SCD PROM | Teresi JA, Kleinman M, Ocepek-Welikson K, Ramirez M, Gurland B, Lantigua R, et al. Applications of Item Response Theory to the Examination of the Psychometric Properties and Differential Item Functioning of the Comprehensive Assessment and Referral Evaluation Dementia Diagnostic Scale among Samples of Latino, African American, and White Non-Latino Elderly. Res Aging [Internet]. 2000 Nov 1 [cited 2022 Jun 23];22(6):738–73. Available from: <https://doi.org/10.1177/0164027500226007> |
| *82877482* | ***Torrealba*** | 2018 | Measure is not a SCD PROM | Torrealba E, Garcia-Morales P, Cejudo JC, Diaz M, Rodriguez-Esparragon F, Fabre O, et al. In-Out-Test: A New Paradigm for Sorting the Wheat from the Chaff in Prodromal Alzheimer’s Disease. JAD [Internet]. 2019 Jan 8 [cited 2022 Jun 23];67(1):265–77. Available from: <https://www.medra.org/servlet/aliasResolver?alias=iospress&doi=10.3233/JAD-171007> |
| *82877489* | ***Van Patten*** | 2018 | Validation of SCD PROM not the aim of the study | Van Patten R, Britton K, Tremont G. Comparing the Mini-Mental State Examination and the modified Mini-Mental State Examination in the detection of mild cognitive impairment in older adults. Int Psychogeriatr [Internet]. 2019 May [cited 2022 Jun 23];31(5):693–701. Available from: <https://www.cambridge.org/core/product/identifier/S1041610218001023/type/journal_article> |
| *82877515* | ***Vannini*** | 2017 | Validation of SCD PROM not the aim of the study | Vannini P, Amariglio R, Hanseeuw B, Johnson KA, McLaren DG, Chhatwal J, et al. Memory self-awareness in the preclinical and prodromal stages of Alzheimer’s disease. Neuropsychologia [Internet]. 2017 May [cited 2022 Jun 23];99:343–9. Available from: <https://linkinghub.elsevier.com/retrieve/pii/S0028393217301239> |
